# Oldest old’s travel mode choice and new mobility technology acceptance: case in America and China

**DOI:** 10.3389/fpubh.2024.1344854

**Published:** 2024-05-02

**Authors:** Yang Zhang, Jinping Guan, Lisa A. D'Ambrosio, Julie Miller, Chaiwoo Lee, Kai Zhang, Joseph F. Coughlin

**Affiliations:** ^1^Harbin Institute of Technology, Harbin, China; ^2^Harbin Institute of Technology (Shenzhen), Shenzhen, Shenzhen, China; ^3^Massachusetts Institute of Technology, Cambridge, MA, United States; ^4^Shenzhen International Graduate School, Tsinghua University, Shenzhen, Guangdong Province, China

**Keywords:** oldest old, travel mode choice, new mobility technology, familiarity and acceptance, American and Chinese samples

## Abstract

**Introduction:**

The oldest olds (aged 85 and over) are the fastest-growing age segment. However, our understanding of their mobility is limited. To address this gap, we invited 19 U.S. and 30 Chinese “oldest old” to take part in focus groups and complete a mobility questionnaire. We focus on travel mode choice, which includes changes in travel modes, frequency of usage, and perceptions of comfort.

**Methods:**

Older adults’ familiarity and acceptance of new mobility technologies (e.g., ridesharing, carsharing, and autonomous vehicles) were measured by questionnaire and focus group. Word clouds were also used to illustrate people’s reasons for choosing their primary mode of transportation.

**Results and discussion:**

The results show that both panels of older adults similarly feel some extent of travel limitations. But the responses among the two groups differ: 18 American participants chose “drive myself” as their primary option a decade ago, while 11 chose it now; no Chinese participants selected it either a decade ago or now. Both currently and 10 years ago, there was a significant difference in mode choice between participants in China and the United States. However, this gap has narrowed over the past decade. Participants in China have significantly changed their transportation preferences compared to 10 years ago, while participants in the US have remained nearly unchanged. American respondents consider “ease” as an important factor, while Chinese respondents pay more attention to “safety” and “no other option to get around” when making travel mode choices. Compared to Chinese participants, American participants were more comfortable with driving an autonomous vehicle. These differences may result from the various developmental stages and transportation policies of the two countries. This study supports the development of new mobility technologies for the oldest old to improve their quality of life.

## 1 Introduction

The world’s older population continues to grow at an unprecedented rate. Today, 8.5% of people worldwide (617 million) are aged 65 and over (1). In New York City, as the Baby Boomers age, the older adult population is growing rapidly. Older adults have increased five times faster than the general population, rising nearly 30 percent since 2000 (2). Likewise, according to the World Health Organization, the aging of China’s population is accelerating (3). From 1950 to 2015, China experienced an increase in life expectancy from 44.6 to 75.3 years. It is estimated that life expectancy could reach 80 years in 2050 (Library Cataloging-in-Publication Data, 2016). Among older adults, the “oldest old” are those who are aged 85 and older (4). On a global scale, by 2030, the population of the oldest old is expected to have increased by 151% since 2005, compared to 104% for those aged 65 and over, and only 21% for those under 65 (United Nations Department of Economic and Social Affairs, 2005), and it is the fastest-growing age segment.

As older people generally do not need to commute to work, their transportation needs tend to shift toward more flexible travel options. Grocery shopping, social visits (friends, relatives), medical care, leisure activities, and volunteering will characterize daily travel (5). However, older adults, especially the oldest old, face challenges with daily travel. Accessibility and mobility are key factors in traveling. As older individuals often face declining health and reduced physical function, they may encounter limitations in mobility (6). This can manifest as difficulties in using traditional public transportation, cessation of driving (7, 8), and encountering transportation challenges that may lead to social exclusion (9). At the same time, age affects driving ability, which can increase traffic risk (10). Crash statistics also indicate that older drivers are more vulnerable in crash situations and pose a risk to themselves and other traffic participants (11, 12).

To address the significant mobility challenges faced by older adults, we can anticipate that new technology-enabled transportation options, such as autonomous vehicles, carsharing, and ridesharing services, could help meet the substantial market demand among older adults. Autonomous vehicles represent a significant application scenario for cutting-edge technologies like artificial intelligence in the automotive industry (13). It has the potential to significantly improve road safety, reduce urban congestion, and enhance urban mobility and public health (14–16). Studies have shown that the public has positive implicit attitudes toward self-driving cars and believes they can expand the range of people’s activities (17–19). Self-driving cars have the potential to excite younger generations and technology enthusiasts, as well as provide more convenient and accessible transportation options for those who find driving challenging as they age. According to Harb et al. (20), self-driving cars may also increase travel among socially active retirees who are becoming less confident in their driving abilities. Carsharing and ridesharing services also represent technology that promises to increase mobility for older adults. Evidence is emerging that many ‘younger old’ individuals are also beginning to experiment with ride-sharing services (21). As such, these new technology-enabled travel modes could cater to a significant market among older adults. However, to date, there is little understanding of how this population, especially the oldest old, would react to these new technologies.

To better understand the transportation challenges faced by older adults, we posed several research questions.

1. What are the differences in travel mode choice and mode choice changes over 10 years in different countries, such as the United States and China?
2. What factors do the oldest old consider when making travel mode choices?
3. With new mobility technologies emerging, such as autonomous vehicles, carsharing, and ridesharing services entering the market, what are the perceptions and acceptance of the older adults toward these new technologies?

To answer these questions, we conducted several focus groups with participants aged 85 and older from the United States and China. We collected questionnaires regarding their travel behaviors and perceptions of new mobility technologies.

The remaining sections of this paper are organized as follows. The following section presents a literature review of previous studies on the travel behaviors of older adults, the reasons related to their travel choices, and their familiarity and acceptance of new technologies. The third section covers the methodology and data. The fourth section presents the statistical analysis and results from the American and Chinese samples. The final section describes the conclusions and implications of the study, offering practical recommendations for transport planners and policymakers.

## 2 Literature review

The world’s population is aging, with the number of the oldest old (ages 85 and older) in particular soaring. In a society that is aging, it is crucial to investigate the needs of older people. However, there is a lack of research on the transportation mobility challenges experienced by individuals aged 85 and older. Research has shown that older adults often experience a decline in travel frequency, an increase in travel time, and a decrease in travel distance with age (22). At the same time, the driving ability of older individuals is also affected by age, which further increases traffic risks (10).

Transportation is an essential need for humans, closely linked to independence, autonomy, and quality of life. Although older individuals typically do not need to commute to work, they still require transportation for activities such as shopping, social visits, medical care, and leisure activities (5). Numerous studies have examined the travel mode preferences of the older adults (not specifically the oldest old). Rahman (23) investigated older adults’ perceptions and preferences for five transportation options. The study found that volunteer drivers were the most preferred mode of transportation for both driving and non-driving older adults. Schmöcker et al. (24) found that older adults without disabilities would use busses and streetcars more. Moreover, research in China found that non-motorized travel decreased with increasing age in the older adults compared with the non-older adults (25). Among the studies that have focused on older adults’ choices of transportation modes, very few have examined the oldest old. In the US, older adults’ travel is nearly 90% car-oriented, with relatively low levels of transit usage (26). Collia et al. (27) found that almost 90% of American older adults (65+) travel by private car, a percentage nearly equivalent to the population of those aged 19–64. In a survey conducted in the United Kingdom, 43 % of drivers aged 60 and over stated that they would cease driving because of health concerns (28). In contrast, Zhang et al. found that in China, walking was the primary mode of travel for older adults, with only 1.58% of the older adults (aged 60 and above) using private cars for transportation (22). Compared with younger individuals, older people in China primarily travel on foot, followed by public transportation and bicycles (29).

Several influential factors affect the choice of primary travel modes among older adults. Zhu and Fan (30) and Sun et al. (31) found that traveling for free purposes, such as community engagement, exercise, and leisure, leads to more positive emotions than commuting trips. In China, research has found that the impact of public transport accessibility and the walking environment on the travel behavior of older people is significant (32). In addition, older people experiencing limited mobility or frailty must navigate additional barriers to public transport use compared to able-bodied riders (33). Other researchers have investigated the effects of urban transportation infrastructure systems, environmental pollution, and energy consumption on the mobility of older adults (34).

The previous literature has an obvious limitation. The vast majority of studies focus on older adults in general (those above the age of 60 or 65). However, individuals aged 60–84 or 65–84 often feel less limited in their daily travel and driving abilities than those who are older. Further, mobility needs continue to change and evolve as people age, and some significant changes may occur later than age 65 (35). Therefore, it is important to pay closer attention to the oldest old group (ages 85 and above) rather than making generalizations based on data from younger individuals (ages 60+ or 65+). Additionally, few have conducted any comparative analysis of the travel behaviors of the oldest old across different countries. Additionally, with the rapid development that has been occurring in China over the past few decades, there have been huge changes in various aspects of China, including transport and mobility (36–40). It is also interesting to conduct a comparative analysis of the travel behaviors of the oldest old across different countries, a topic that few studies have explored.

Many older adults are not familiar with common travel services or applications. For instance, in Illinois, many older adults were unfamiliar with the public transportation system and did not consider it as an alternative to driving. Policies hold promise to preserve and enhance the safe mobility of older adults (41). Several studies show that older drivers use new technologies differently from younger drivers (42–44). More studies could be conducted among the oldest old.

The acceptance of new technology among older individuals has significant implications for their mobility. Generally, older adults exhibit a lower level of acceptance of new mobility technology. The older the person is, the less likely he or she will use the new mobility technology, such as autonomous vehicles, which was found in the United States, United Kingdom, the Netherlands, and Australia (45–48). In China, older participants are unwilling to pay more for autonomous vehicles (49). In addition, a study found that 26% of the respondents who are 65 years old and older prioritize flexibility first, followed by 14% who prioritize reliability, 13% who prioritize cost, 13% who prioritize speed, and 12% cite safety as first (50). Age is always a key factor in these analyses. However, when it comes to the oldest old, very few studies have focused on this population aged 85 and above. People who are 85 years old and older face more mobility challenges than younger adults aged 60 to 75, which may lead to different attitudes toward new mobility technology. Therefore, it is necessary to study the acceptance of new mobility technologies among the oldest old; however, there are very few studies that have done so.

In summary, there are four trends worth noting.

1. The trend toward an aging population: Globally, the proportion of older people is growing rapidly, and the “oldest old” group, aged 85 and over, is of particular concern, as their mobility needs and challenges are likely to be more complex than those of other age groups.
2. Importance of travel for older adults: It is crucial to acknowledge the importance of travel for older individuals as it significantly contributes to their quality of life, social engagement, and independence. As individuals age, they may encounter travel limitations that directly affect their daily lives and well-being.
3. Development of new mobility technologies: With the emergence of new mobility technologies such as self-driving vehicles and shared mobility services, it is particularly important to examine how these technologies affect the travel choices and acceptance of the oldest old. These technologies have the potential to enhance travel conditions for older individuals. However, there is also a need to understand their acceptance and willingness to use them.
4. Lack of intercultural research: There has been limited research conducted on travel among older adults in diverse cultural contexts. Exploring the impact of cultural differences on travel choices by comparing the travel behavior of older people in China and the United States.

Therefore, findings from this study would help to fill the research gaps and also support us in better understanding and meeting the mobility needs of the oldest old.

## 3 Research methodology

The study was designed to explore similarities and differences between the oldest individuals in America and China, focusing on their travel-related challenges, shifts in habits, and attitudes toward new technologies. To figure this out, we collected a small number of samples in both China and the United States. Detailed information is separated into “Participants” and “Procedures and Instruments” as follows.

### 3.1 Participants

In this study, a total of 49 ‘oldest old’ participants completed the survey, including 19 Americans and 30 Chinese. In the United States, a focus group of 19 participants was convened in December 2015 at the MIT Age Lab to discuss travel-related issues. This was part of a bi-monthly panel study of the oldest old, where participants contribute to research on a series of topics related to aging (51, 52). All of the American participants were white and lived in the metro Boston area (MA, United States). Ten of them were women. Data for the Chinese samples was collected in August 2018. Participants were residents living in either Shanghai (a province-level city) or Zhejiang (province), eastern China. Fifteen of them were women. Most American and Chinese participants in this study were much healthier, wealthier, and had a higher level of education than the local average, so due to the small sample sizes and their lack of representativeness, the study is not generalizable. In addition, data for the US sample were collected in 2015, while data for the Chinese sample were collected in 2018. So, a time difference exists, and the results of this study can only represent the period from 2015 to 2018.

It is worth mentioning that due to the correlation between advanced age and declining health and physical function, which results in decreased activity levels, it is challenging to locate individuals aged 85 and above in real-life social surveys. Additionally, individuals who fit this description are often not easily motivated and may be hesitant to participate in complex research processes, such as completing questionnaires or engaging in focus groups. This reluctance makes sample collection more challenging.

### 3.2 Procedures and instruments

To enhance the diversity of the sample, the criteria used for selecting participants in the study were: (1) aged over 85 years and (2) having a permanent residence in either China or the United States. The study utilized convenience sampling and snowball sampling methods. The sampling was divided into an online and an offline component. For the offline component, we initially targeted readily available and accessible individuals. We began with one person and expanded the sample as participants referred others. If there were eligible participants, we sent emails describing the key elements and process of the study and gave them the option to participate to ensure they were all volunteering. For the online component, we post recruitment information on the recruiting website and WeChat to attract potential volunteers.

In terms of data collection, the primary instruments used in the study were questionnaires (53) and focus groups (54). During the study, participants first completed a questionnaire consisting of 44 questions (refer to Table 1). The questions mainly focused on: (1) limitations in travel mode choices and willingness to adopt new technologies to overcome these limitations; (2) utilization of assistive devices; (3) frequency of using different modes of transportation such as various vehicles, public transportation, bicycles, or walking, along with the reasons for choosing these modes; and changes in travel mode preferences from 10 years ago to the present. Question formats were predominantly Likert scale, combined with some single-choice, multiple-choice, and open-ended questions. To validate the results, we tested the reliability of the questionnaire in the contexts of China and the United States using Cronbach’s alpha reliability coefficients. The results for China and the United States were 0.805 and 0.814, respectively, indicating good consistency of the questionnaire scale. The content validity of the questionnaire was evaluated by five experts, and the Item-Content Validity Index (I-CVI) for all questions used in the experiment was 1.00. This indicates that the questionnaire scale was in alignment with the content of the examination. However, due to the small sample size, conducting exploratory and validation factor analyses for the structural validity of the questionnaire was not feasible.

**Table 1:** Summary of the survey questions in the questionnaire.

Question category	Content
Demographics	Age, gender
Travel limitations and assistive devices	Capacity for mobility, scenarios for using assistive mobility devices, frequency of using assistive mobility devices, types of assistive mobility devices used
Travel mode choice	Frequency of common travel modes, types of vehicles used for driving, accessibility to public transport, primary travel mode, evaluation of primary travel mode, age for adopting current transportation habit, travel mode choice 10 years ago
Acceptance of new mobility technology	Evaluation of comfort across different travel modes, familiarity with travel services and applications, preference of primary travel mode

Following the questionnaire, a lecturer provided specific information to the oldest old regarding existing tools to assist with mobility. Finally, researchers utilized small focus groups as a qualitative research method to allow older adults to discuss the following: (1) personal experiences with various modes of transportation; (2) knowledge about current transportation services and applications; (3) feelings regarding the use of current and previous primary modes of transportation; and (4) suggestions for improving older adults travel. To ensure the credibility of the focus groups, we validated the results of the discussions using multiple data collection methods such as interviews, observations, and document analyses. This approach yielded more consistent results. Additionally, we meticulously documented the discussion process and transcribed it accurately to ensure the consistency of the data during subsequent analysis. Regarding validity, we also assessed the comprehensiveness of the discussion guide through expert review to ensure that it addressed all the key aspects of the research questions.

The statistical analysis of the questionnaires in this study involved descriptive statistics and correlation analysis, including a t-test. The focus group data was analyzed using content analysis, and word clouds were utilized to visually represent participants’ tendencies more intuitively. Statistical analysis was conducted in SPSS (version 26.0) using the appropriate tools. To analyze travel mode frequencies, values are assigned to different frequency gradients, and frequency means are calculated to compare the frequency of different modes of travel for participants in different countries.

## 4 Statistical analysis and results

### 4.1 Travel limitations and assistive devices

In our study, many participants felt limited in their daily travel. Of 19 American respondents, eight indicated that they were limited in what they could do or the places they could go because they lacked transportation options to get there, while seven disagreed and four remained neutral. In the Chinese sample, 11 agreed they were limited, while 10 disapproved and nine were neutral. Although not representative, participants’ answers indicated that people experienced limitations in their daily travel mode choices because of a lack of transportation options. We conducted an independent samples t-test for selection on assisted mobile devices in China and the United States (Table 2), and the results showed that the selection is not significantly different in the two countries, suggesting these older American and Chinese participants felt a similar extent of traveling difficulty.

**Table 2:** Independent samples t-test for assisted mobile device selection.

	China (*n* = 30)		America (*n* = 19)		t-test for equality of means	
	Mean	Std. deviation	Mean	Std. deviation	t	*p*
Use of assistive devices	2.32	1.757	1.86	1.062	−1.248	0.217

Variable coding:1-I do not use a mobility device, 2-Cane, 3-Walker, 4-Motorized scooter, 5-Motorized wheelchair, 6-Wheelchair.

**Figure 1:**
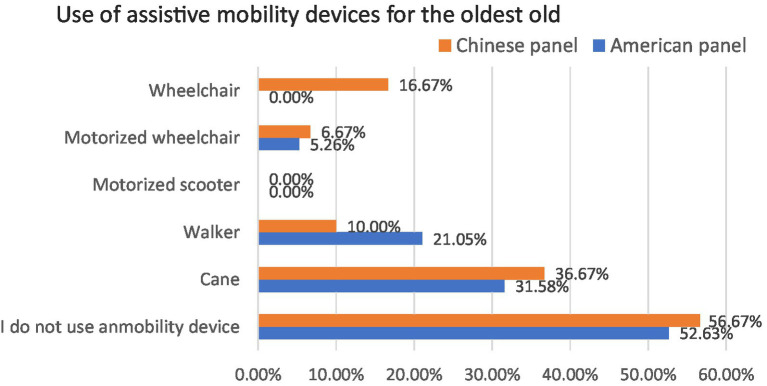
The current use of assistive mobility devices.

Regarding assistive mobility devices, both American and Chinese participants reported canes and walkers as their most frequently used travel assistive tools. However, in the Chinese panel, five of the participants used wheelchairs while none of the American respondents reported wheelchair as their choice (Figure 1).

### 4.2 Travel mode choice comparison and its change over the years

**Figure 2:**
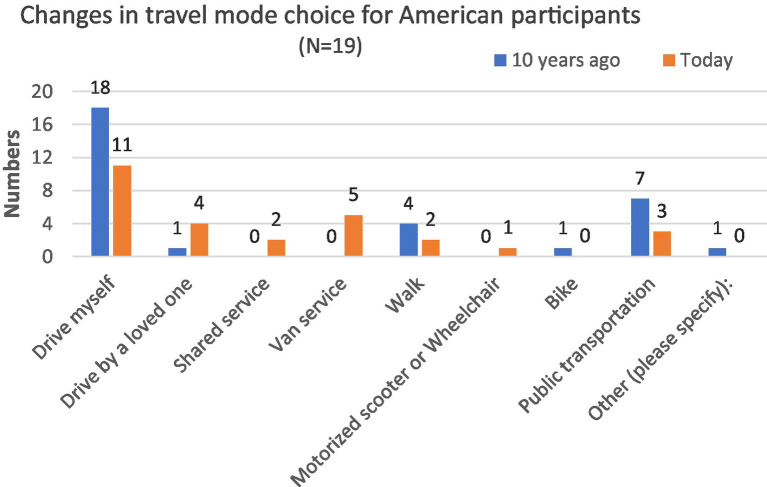
Changes in transportation modes, US respondents ages 85 + .

**Figure 3:**
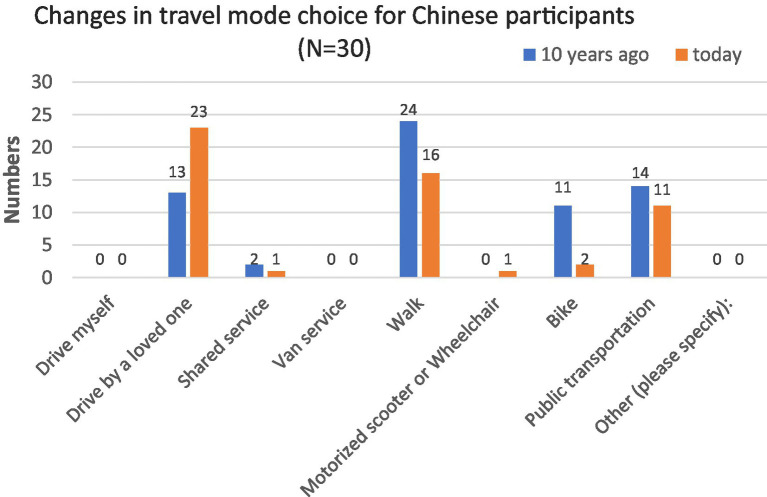
Changes in transportation modes, Chinese respondents ages 85 + .

Respondents were asked in the questionnaire about their current and previous (10 years prior) travel modes, and there were noteworthy differences across time and location. Figures 2, 3 show that the current and past transportation modes participants chose diverged over time. Among US participants, 18 chose to drive themselves one decade ago while 11 chose to drive currently. Similarly, the number of participants who traveled by public transportation declined from seven to three, even though half of the American participants (*N* = 9) stated that it took them no more than 15 min to walk from their home to the nearest transit station. American respondents who preferred being driven by others (including by a loved one, shared service, or van service) increased from a decade ago to the present. This is consistent with previous studies that found the private car is the primary travel mode for older adults, and 89.3% of those ages 65 and older meet their daily travel needs by private vehicle (27).

As for the Chinese sample, none of the participants chose to drive themselves or use van service one decade ago or at present (Figure 3). Ten years ago, most Chinese participants’ choices included walking (*N* = 24), public transportation (*N* = 14), being driven by a loved one (*N* = 13), or riding a bike (*N* = 11). Currently, most Chinese participants reported they traveled by being taken by a loved one (*N* = 23), walking (*N* = 16), and taking public transportation (*N* = 11). More Chinese participants (70%; *N* = 21) responded that it would take them no more than 15 min to walk from home to the nearest transit station. This may explain why there were more Chinese than American respondents who chose walking or public transportation as travel mode choices. Overall, the differences in travel between Chinese and American participants are evident both now and 10 years ago, as shown by the data in Table 3. The two panels do, however, share some similarities about their travel mode choices: shared car service, van service, motorized scooters, or wheelchairs were not popular among participants (Figure 3).

**Table 3:** Independent samples t-test for different times on Chinese and American samples.

	China (*n* = 30)		America (*n* = 19)		t-test for equality of means	
	Mean	Std. deviation	Mean	Std. deviation	t	*p*
Today	4.39	2.375	3.04	2.317	2.467	0.016*
10 years ago	5.33	2.138	3.5	3.152	2.958	0.005**

Variable coding: 1-Drive myself, 2-Drive by a loved one, 3-shared service, 4-Van service, 5-Walk, 6-Motorized scooter or Wheelchair, 7-Bike, 8-Public transportation, 9-Other. ***p-value < 0.001. **p-value < 0.05. *p-value < 0.1.

Among American respondents, a declining number of people driving themselves and using public transportation could be explained by the fact that challenges associated with aging. Chinese respondents also encounter the same problems which could be proved with an increasing number relying on being driven by a loved one and declining numbers of people who report walking or biking.

Table 3 shows a significant difference in transport travel choices between participants in China and the United States, both presently and 10 years ago. However, this gap has decreased over the past decade, which is consistent with the information presented in Table 4. While there has been a notable shift in transport travel choices among participants in China over the 10 years, there has been no such change among participants in the United States.

**Table 4:** Independent samples t-test for Chinese and American samples at different time.

	Today		10 years ago		t-test for equality of means	
	Mean	Std. deviation	Mean	Std. deviation	t	*p*
China (*n* = 30)	4.39	2.375	5.33	2.138	−0.26	0.026*
America (*n* = 30)	3.04	2.317	3.50	3.152	−0.655	0.515

Variable coding: 1-Drive myself, 2-Drive by a loved one, 3-shared service, 4-Van service, 5-Walk, 6-Motorized scooter or Wheelchair, 7-Bike, 8-Public transportation, 9-Other. ***p-value < 0.001. **p-value < 0.05. *p-value < 0.1.

According to the focus group, American and Chinese respondents differed significantly around the age at which they started to use their current primary means of transportation. More than 60% of American participants (*N* = 12) stated that they began to use their primary mode in their 20s or earlier, while the rest said in their 70s or later. However, only 30% of Chinese participants responded that they adopted their current traveling modes in their 30s or earlier, while others said that it was in their 50s or later. As a result, the travel choices of US respondents have remained nearly the same over the past decade. Chinese respondents were more likely to report a change in mode of transportation over their life span than were American participants. This may be related to the rapid pace of urbanization and development in China. Compared to more developed countries like America, China is currently in a fast-developing phase, leading to significant changes in the travel choices of Chinese participants over the decade.

### 4.3 Diverse reasons for choosing primary transportation modes currently and one decade ago

The reasons why people chose their modes of transportation varied a great deal not just across the two samples but also over time (Table 5). A decade ago, American respondents considered “convenience” and “accessibility” as the most important factors why they chose their primary means of transportation, but currently “ease” is the most important one. “Safety” was not crucial for American respondents. Among Chinese respondents, however, “convenience” and “safety” were of great importance in participants’ consideration a decade ago, while “safety” and “reliability” were more prominent at present. The greater attention among Chinese participants to “safety” may indicate that they hanker for safer traveling environments, while American participants may not have considered “safety” as vital because they live in a more developed country and experience a greater sense of safety. We know from other data/sources in the literature that older adults in the US are concerned about safety and security when they make transportation choices—but for this relatively mobile and affluent sample, safety and security may have been less of a factor in the decision around mode choice.

**Table 5:** Word cloud of reasons for choosing primary means of transportation.

	Ten years ago	Today
American responses	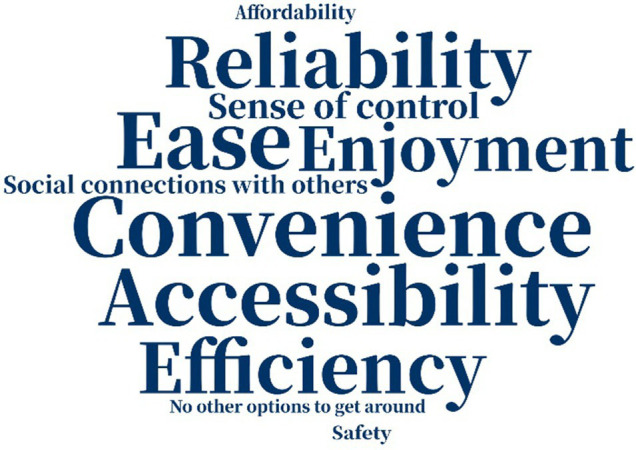	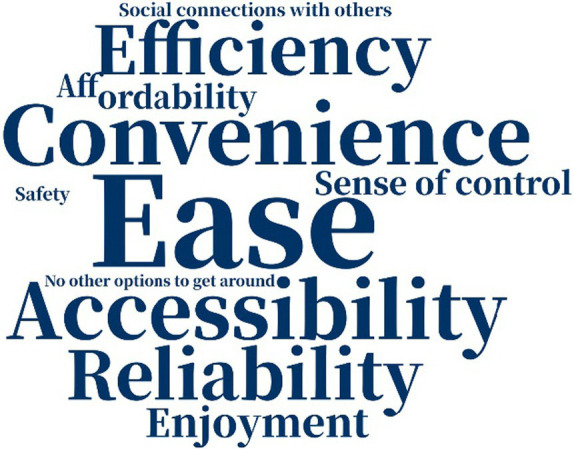
Chinese responses	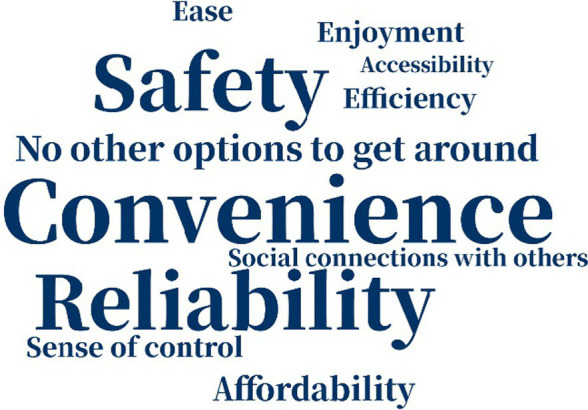	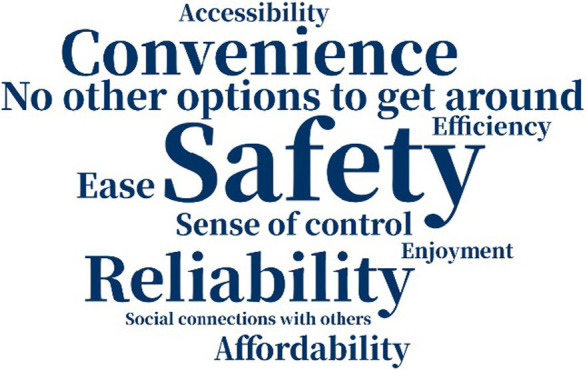

The size of the words in the graph is proportional to the frequency of their appearance in the focus group, with elements of greatest concern to older people being more visually prominent.

“No other option” to get around also figured into older adults’ choice of mode: one of the 19 American respondents and eight of the 30 Chinese respondents indicated that they used the mode they did because they had “no other options to get around.” This suggests that compared to the American sample, Chinese participants felt more restricted in their travel mode options, and “accessibility” and “sense of control” were less important factors in mode choice. The choice of different factors indicated that American participants might be more likely to value efficient and enjoyable traveling, while Chinese participants weighted safety and reliability more highly in their choice of mode, partly because of no other available options.

### 4.4 The frequency of use of different transportation modes

Table 6 presents differences in the frequency of use of different transportation modes among American participants (*N* = 19) and Chinese participants (*N* = 30). More than 90% of American and 70% of Chinese respondents chose “never” to ride a bike—perhaps reflecting their age. Among American participants, however, a majority indicated that they drove a car almost every day. For this American sample, who live in a metro-urban area—and of whom half live within 15 min of the nearest public transit stop, public transportation was also an important transportation mode. Among Chinese respondents, the ratios of “never” riding a bike and “never” driving were much higher than in the American group. Moreover, the frequency of taking public transportation was not high, with only 50% of Chinese participants reporting that they chose to take public transport only “a few days a week” or “a few days a month.” In contrast, walking “every day” was chosen by 60% of Chinese participants. The frequency averages indicate that Chinese participants ride bicycles more frequently than American participants. Additionally, there is a significant disparity between the two countries in terms of walking and driving. Chinese participants are more likely to walk and rarely drive, whereas American participants are less likely to walk and driving is more common.

**Table 6:** Descriptive statistics of transportation modes frequency.

	Ride a bike		Walk		Public transportation		Drive a car or other motor vehicle	
	American (*n* = 19)	China (*n* = 30)						
Never	95%	73%	32%	0%	11%	37%	21%	73%
A few times a year	0%	3%	11%	13%	11%	12%	11%	13%
A few days a month	0%	3%	16%	10%	17%	30%	0%	10%
A few days a week	5%	7%	11%	17%	39%	20%	11%	3%
Almost every day	0%	13%	32%	60%	11%	3%	58%	0%
**Frequency mean**	0.15	0.82	**2.04**	**3.24**	2.06	1.44	**2.76**	**0.42**
Total	100%	100%	100%	100%	100%	100%	100%	100%
	Data of America	Data of China						

*n*% = (responded number/overall group sample)*100%. Variable coding of **Frequency mean**: 0-Never, 1-A few times a year, 2-A few days a month, 3-A few days a week, 4-Almost every day.

Although the study samples are not representative of the oldest old in either the U.S. or China, the results do suggest that American older adults are more likely to access convenient traveling modes and to be able to choose a mode they prefer compared with Chinese participants. For example, within this sample, American participants would probably not be too worried about parking fees or fuel costs, while Chinese participants were more likely to choose to “walk” and “ride a bike” which are cheaper or even free modes. Although both the US and Chinese samples are urban ones, the Chinese samples are in a more populous and dense urban area. In general, the costs of the modes across the two contexts are likely to be very different.

### 4.5 Perception of the comfort of various transportation modes

Although efficiency is a crucial factor when choosing modes of transportation, comfort is also important. Almost 80% of the American sample (*N* = 15) agreed that they felt comfortable driving a regular vehicle, and 12 strongly agreed they were comfortable doing so. More than half of the American sample said they would feel comfortable taking public transportation, such as the bus, subway, or train. Also, most of them would feel uncomfortable walking though, thus few of them take walking as an efficient way. A majority—70%—of American respondents strongly disagreed that they would feel comfortable using a bike, and 50% of them were averse to using a motorized scooter or wheelchair to get around. In contrast, 70% stated that they felt comfortable taking flights.

The Chinese sample presented differently concerning comfort using different transportation modes. First, about 50% of them reported that they did not feel comfortable driving a regular vehicle. Second, although more than half of Chinese participants stated they felt comfortable taking public transportation modes such as the bus, subway, or train, around a third (*N* = 10) were strongly averse to using these modes. Moreover, feeling comfortable walking for 15 min or longer (*N* = 22, ration = 73%) is consistent with the situation that most participants choose walking as their primary mode in Figure 3. Riding a bike, or using a motorized scooter or wheelchair was not popular among the Chinese sample. Finally, most Chinese respondents indicated that they were not comfortable taking flights.

### 4.6 Acceptance of new technologies

#### 4.6.1 Familiarity with common traveling services and applications

Unlike most stereotypes of older adults, especially the oldest old, most in the American sample were willing to accept new technology-enabled tools to help with their traveling needs; the sample was not true in the Chinese sample. More than 70% of the American respondents (*N* = 14) agreed that they could learn to use new technologies quickly, while in the Chinese group, only 10% agreed (*N* = 3) and 80% disagreed (*N* = 24). In short, American participants believed they could access and learn to use the latest technologies while Chinese participants did not, even though these two groups—although in different countries—shared similar health and social conditions and comparable educational backgrounds.

As shown in Table 7, the majority of respondents from both countries were aware of common transportation applications. Usage of different applications varied, however. Online maps were used by most American participants (79%), and GPS navigation systems were used by a plurality. Fewer American participants had used ridesharing services (21%), car-sharing services (5%), or parking information (5%), but these applications were still familiar to a majority. Among the Chinese sample, most participants knew of these different technologies, but relatively few of them had used any of them.

**Table 7:** Descriptive statistics of familiarity with common traveling services and applications.

	American sample (*N* = 19)			Chinese sample (*N* = 30)			Total (*N* = 49)		
Application type	Use or have used	Know, but not used	Do not know this	Use or have used	Know, but not used	Do not know this	Use or have used	Know, but not used	Do not know this
Bus/Subway tracking	5(26%)	6(32%)	6(32%)	3(10%)	22(73%)	5(17%)	8(16%)	28(57%)	11(22%)
GPS navigation	9(47%)	8(42%)	0(0)	3(10%)	22(73%)	0(0)	12(24%)	30(61%)	0(0)
Online maps	15(79%)	2(11%)	1(5%)	5(17%)	18(60%)	1(3%)	20(41)	20(41%)	2(4%)
Parking information system	1(5%)	13(68%)	4(21%)	4(13%)	19(63%)	0(0)	19(39%)	32(65%)	4(8%)
Ridesharing service	4(21%)	12(63%)	2(11%)	3(10%)	21(70%)	2(7%)	7(14%)	33(67%)	4(8%)
Car sharing service	1(5%)	15(79%)	1(5%)	4(13%)	17(57%)	0(0)	5(10%)	32(65%)	1(2%)

*n*% = (responded number/overall group sample) *100%.

#### 4.6.2 How do you feel when using new technologies such as autonomous vehicles?

The questionnaire and focus groups also asked participants whether they felt comfortable using new technologies, including future applications. More than half of US participants (*N* = 10) agreed that they would feel comfortable driving an autonomous vehicle. Among the ones who are not so into autonomous vehicles, one of the US participants in the focus group mentioned: “I enjoy driving my own car and I am used to it. Why do I need it to be self-driving? Some driving assistance is good.” Among the 21 Chinese responses (nine responses missing), none of them showed significant interest in the new technology. More than 60% of them (*N* = 13) mentioned they would feel comfortable neither driving a regular vehicle nor driving an autonomous car.

The responses around the willingness and ability to learn new technologies and the answers to future applications such as in autonomous vehicles are consistent. The high interest in and desire for new technologies among American participants may also be attributed to the extent of development in America, while Chinese respondents may be more likely to live a “safe” and “steady” life.

These data indicate that the American panel is much more likely to drive themselves to get around. However, compared with younger drivers, older drivers are more likely to contend with physical challenges, such as issues with posture, lack of flexibility, and slower reaction time that may have an impact on the safety of the drivers themselves as well as pedestrians and other road users.

## 5 Discussion and conclusion

Mobility is key to a person’s life. The population of the oldest old, those aged 85 and over, is growing rapidly. However, little attention has been paid to their mobility needs, which could restrict their activities and access to various destinations.

This study analyzed data from 19 American and 30 Chinese “oldest old” participants. The analysis was based on their participation in focus groups and questionnaire responses regarding their personal experiences with and recognition of various types of transportation modes and new technologies, including autonomous vehicles, carsharing, and ridesharing services. The study aimed to investigate differences in travel-related issues, changes in travel habits and mode preferences, as well as familiarity and comfort with recent travel technologies among the oldest old in various countries.

### 5.1 Contribution to existing literature

The importance of this study to the existing literature lies in four main areas. (1) Specific population focus: The study focuses on the population of the “oldest old,” an age group that is often overlooked in various types of research. The specific focus on this population fills an important gap in gerontological research and represents a significant step toward a more comprehensive and empathetic understanding of the complexities associated with advanced age. (2) Intercultural comparison: The study compares the travel mode choices and acceptance of new mobility technologies among the “oldest old” samples in the U.S. and China, providing insights into intercultural perspectives. (3) Attitudes toward new mobility technologies: The study investigated the attitudes and acceptance of the “oldest old” toward emerging mobility technologies (e.g., self-driving vehicles, shared mobility services). This exploration is crucial for understanding the potential utilization of these technologies among older adults. (4) Travel choices: This study provides insights into the differences in travel mode choices among the “oldest old” and compares potential reasons for selecting the primary mode of travel.

### 5.2 Conclusion of the study results

The results of the analysis indicate that Chinese and American participants encounter the same dilemma when it comes to traveling. American participants feel they have more travel mode options than Chinese participants. Although 50% of American participants stated that it took them no more than 15 min to walk from their home to the nearest transit station, most of them still chose to drive themselves. Compared to their mode choice a decade ago, American respondents still preferred driving, although more now favored being driven by others (including by a loved one, shared service, or van service).

Additionally, the analysis indicated the importance of establishing “convenient” and “easy” transportation options for the oldest old to travel outside of their homes. For both American and Chinese respondents, shared service, van service, and motorized scooters were listed as options but were not widely used. Among Chinese respondents, none chose to drive themselves or use a van service. Walking, public transport, and being driven by others were the most popular modes of transportation. Participants were more likely to focus on “safe” and “reliable” travel modes, while “accessibility” and “sense of control” were less important. Due to the size and nature of the sample, it is not practical to use a regression model to address questions about the factors influencing the travel mode choice of the oldest old. Instead, we utilized a word cloud methodology, drawing on interview responses to investigate the reasons individuals provided for their mode choices.

Regarding the differences in transportation between participants in the US and China, both presently and 10 years ago, the contrast is evident. This difference may be attributed to the significant variance in habits and culture between the two nations, as reflected in the percentage of individuals who drive alone. However, this disparity has decreased over the past decade. In a 10 year comparison of Chinese and American participants, there was a significant change in the transportation travel choices of the Chinese participants, while the American participants showed almost no change.

As for the attitudes of the oldest old toward new mobility technologies, many participants in the American sample defied stereotypes by expressing their willingness to embrace technology-enabled tools to assist with their travel needs. In contrast, Chinese respondents seemed to be less inclined to do so. More than 70% of American participants reported that they believed they could quickly learn to use new technologies. However, when it came to newer travel services and applications, usage varied. Few American participants had used ridesharing services, carsharing services, or parking information, but these applications were still familiar to the majority. In the Chinese sample, most participants were aware of common applications, but only a few of them actually used or had used them. Although the oldest individuals might be the primary beneficiaries of these new technologies, these results suggest that transportation service providers and developers may need to make more efforts to promote their new products and services and ensure they are easily accessible to this population.

### 5.3 Significance of the Study

Three implications can be drawn from the findings of this study to assist policymakers and planners in better supporting the mobility of the oldest old.

First, differences in the choice of and preference for travel modes may result from different development stages—for example, developed versus developing countries. According to the International Monetary Fund, in 2018, the GDP *per capita* in the US was seven times that of China. The U.S. is classified as a developed country, while China is considered a developing country. The variation in development levels could imply that American participants have the luxury to prioritize the quality of their transportation experiences. When questioned about their mode change or selection reasons, they highlighted “convenience,” “accessibility,” and “ease.” In contrast, respondents from the Chinese sample were more likely to report “material cost” and “safety” as their primary considerations regarding the choice of mode. One can scarcely feel happy if their basic needs are not being met. In this context, American participants were more likely to offer reasons for their choices that reflected higher needs such as “self-actualization,” which includes seeking emotional enjoyment. The results also suggest that Americans adopted their preferred travel modes earlier and used them for a longer duration compared to the respondents from the Chinese sample. Over 60% of American participants stated that they adopted their travel mode in their 20s or earlier, while the rest said they did so in their 70s or later. However, only 30% of Chinese participants responded that they made their travel mode choices in their 30s or earlier. These differences may result from greater changes in China over the past several decades as it has rapidly developed (36–40), in contrast to the U.S.’s slower, steadier growth path as a more developed country. China’s evolving development may also lead people to focus more on “cost” and “safety” as important factors in their choices, while in the more developed U.S., respondents could give more weight to “convenience” and “accessibility.”

A second reason for the differences between the two samples could be related to the distinct transportation infrastructures and mobility environments in which each resides. Compared to the highly developed railway system in China, the railway system in the U.S. is less developed. For many American travelers, taking a train is much less convenient than driving themselves or taking a flight (27). Most Chinese participants had never flown in an airplane, partly due to the relatively high prices in China. Additionally, most reported that they would feel uncomfortable using this mode of transportation. Thus, it is crucial to consider the transportation preferences and infrastructure differences in different countries when developing improved transportation services for the oldest old.

Thirdly, new technologies could play a critical role in supporting older adults’ mobility. Transportation agencies should explore utilizing the internet to disseminate information to older adults regarding their mobility options. They should also consider leveraging new tools to enhance older adults’ comfort levels in learning about and accessing various mobility options and support services. Additionally, to enhance the mobility of individuals aged 85 and above, several alternative approaches can be considered. We can enhance the physical and mental well-being of older individuals to boost their desire to travel and ensure environmental accessibility to facilitate their journeys. Transport services can also be customized to meet the needs of older individuals, and urban planning can incorporate more spaces suitable for people of all ages. Moreover, inclusive urban planning incorporates design principles that prioritize accessibility for people of all age groups. Initiatives on environmental accessibility, such as adapting urban environments and taking action to make public spaces more friendly and safe, could facilitate travel for the older adults. In terms of education and training, it is advisable to provide educational programs on assisted mobility technologies and available transportation services. Additionally, training the older adults on how to safely and efficiently use new transportation technologies is essential.

This paper presents a comparative analysis of the travel behaviors of small samples of the oldest old from the U.S. and China over a period of 10 years. The analysis focuses on their choice of travel modes, frequency of usage, and perception of comfort. Although the nature of the data limits generalizations that can be drawn from it, the results highlight the importance, across different countries, of focusing on the transportation transitions and needs of the global population segment of people aged 85 and older. While people’s experiences and transportation environments may vary across different countries, they all share a common need to ensure mobility throughout their lifespans as life expectancy increases, in order to help maintain a high quality of life.

### 5.4 Limitations of the study and further steps

There are still limitations to the present study, including the small sample size resulting from the difficulty in recruiting an extreme population group (the oldest old) and data imbalance between the two samples. The oldest old are often excluded from much data collection and research in practice, partly due to challenges in accessing them. Therefore, these experimental results cannot be generalized to the entire population. In addition, the data used in this study is limited to the year when the survey was conducted, which may also affect the generalizability of the results. The analysis and results presented here also underscore the significance and necessity for further research on this expanding segment of the global population. As a result, this study serves as an initial exploration to fill the research gap on the travel mode choices of the oldest old and their acceptance of new mobility technologies.

For the next stage of the investigation, future studies can address: (1) Expand the sample size by broadening the sampling range and using a more systematic method to improve the universality, representativeness, and stability of the results. (2) Enhance the diversity of the sample by investigating individuals from various regions, cultural backgrounds, economic statuses, and genders to understand the effects of different variables on the travel patterns of the oldest old. (3) Long-term tracking study: Conduct a long-term tracking study to observe how the travel habits of the oldest seniors change over time in relation to health conditions and technological developments.

## Data availability statement

The data underlying this article cannot be shared publicly due to the privacy of individuals who participated in the study. The data will be shared on reasonable request to the corresponding author.

## Author contributions

YZ: Formal analysis, Investigation, Methodology, Software, Visualization, Writing – original draft. JG: Conceptualization, Data curation, Formal analysis, Funding acquisition, Investigation, Methodology, Resources, Validation, Writing – review & editing. LD’A: Conceptualization, Data curation, Investigation, Methodology, Resources, Validation, Writing – review & editing. JM: Writing – review & editing, Data curation, Investigation, Methodology. CL: Writing – review & editing, Data curation, Investigation. KZ: Writing – review & editing, Formal analysis, Resources, Validation. JC: Writing – review & editing, Conceptualization, Funding acquisition, Methodology, Project administration, Resources, Supervision.
